# Endoscopic iso-pathlength self-calibration for direction-resolved retrieval of tissue optical properties

**DOI:** 10.1117/1.JBO.31.6.064305

**Published:** 2026-03-21

**Authors:** Natanel Ovadia, Hamootal Duadi, Dror Fixler

**Affiliations:** a Bar Ilan University, Faculty of Engineering and the Institute of Nanotechnology and Advanced Materials, Ramat Gan, Israel; b Tel Aviv University, Jan Koum Center for Nanoscience and Nanotechnology, Department of Biomedical Engineering, Faculty of Engineering, Tel Aviv, Israel

**Keywords:** iso-pathlength point, endoscopy, Monte Carlo simulation, phantoms, chicken breast, tissue optical properties

## Abstract

**Significance:**

Medical examination of human tissue is preferably performed by imaging the tissue surface. Optical imaging techniques are limited by low penetration depth due to high tissue scattering, whereas sensing techniques can detect changes deeper inside the tissue. Near-infrared sensing methods such as oximetry and fNIRS are already used clinically but have not yet been applied in endoscopy.

**Aim:**

We investigate the existence of iso-pathlength (IPL) points in endoscopic geometry, with the goal of extending the concept of IPL points from cylindrical and half-infinite geometries into hollow cylindrical tissue relevant to endoscopy. In addition, we demonstrate the ability to extract the absorption properties of a tissue at this structure by the IPL and demonstrate it by *ex vivo* experiment.

**Approach:**

The IPL point is a unique position in the full scattering profile, independent of tissue scattering and dependent only on the tissue absorption and geometry. We studied two directions in cylindrical endoscopic geometry: azimuthal and longitudinal. First, diffusion theory with extrapolated zero-boundary conditions was applied to predict IPL positions. These predictions were then tested using Monte Carlo simulations of photon distribution and validated experimentally using phantoms with cylindrical air holes measured by endoscopy. Finally, using the experimentally identified IPL point and applying the same procedure to a standard phantom, a hemoglobin–agar phantom, and chicken breast tissue, we were able to estimate the absorption coefficient of the chicken tissue.

**Results:**

Both azimuthal and longitudinal IPL points were identified. The experimental azimuthal IPL point was found at an angle of 144  deg±3  deg, whereas the longitudinal IPL point appeared at a distance of 0.33±0.05  cm from the laser spot center. These findings confirm the theoretical and simulation predictions. Moreover, from the *ex vivo* experiment of a chicken breast, the IPL point enables us to calculate the absorption coefficient and get $\mu_{\text{a}}=0.94\;{\mathrm{cm}}^{-1}$, within the range of $0.2\;{\mathrm{cm}}^{-1}\leq \mu_{\text{a}}\leq 2\;{\mathrm{cm}}^{-1}$.

**Conclusions:**

The demonstration of IPL points in endoscopic geometry provides a new framework for depth-resolved optical sensing in hollow cylindrical tissues. This approach may enable self-calibrated absorption measurements and open the way for improved diagnostic tools in the digestive system, esophagus, and other hollow organs where conventional endoscopy lacks depth information.

## 1 Introduction

Endoscopy is an important imaging method for examining hollow internal organs such as the esophagus, the stomach, and the colon, developed by Dr John Macintyre in 1894. Today, there are many innovations in the field of endoscopy, which deal with improving the resolution of photography and the detection of various diseases and different ulcers in the internal organs. For example, narrow band imaging (NBI)^1^^,^^2^ endoscopy technique is based on the fact that the light absorption of blood vessels is stronger at the green and blue wavelengths, and therefore, by replacing the classic white light source in the endoscope with a light source based on those colors only, a higher contrast is obtained between the tissue and different blood vessels, facilitating the detection of tumors.^3^ Another example of advanced technology in this field is magnetic controlled capsule endoscopy (MCCE), which allows high-resolution pictures with high reliability.^4^ There are even developments such as virtual colonoscopy (VC) that help create virtual 3D models of the organs.^5^ Even more, in the case of VC, the use of an endoscope is redundant, and the model is achieved using a computed tomography (CT) scanner; hence, it is also called CT colonography.

Despite all of those innovations, pictures obtained from the endoscope are limited, and in most cases, a biopsy of the suspected area is required to establish a definitive diagnosis. For example, in VC examination, polyps with a diameter between 2 and 10 mm can be misdetected by the CT scanner. Moreover, even if the resolution of the endoscope is extremely high, the endoscope is unable to get any information about the tissue depth.

In this work, we present a new sensing technique based on the existence of an iso-pathlength (IPL) point in hollow cylindrical tissue. When measuring tissues with unknown scattering and absorption, the IPL point is a unique geometrical position that allows self-calibration. Though we received some indication from simulations of the existence of this phenomenon in cylindrical geometry,^6^ we have yet to measure the existence of two separate points for the different axes of the cylindrical geometry, azimuthal, and longitudinal. In this work, we examined the existence of these two IPL points and proved, in simulation,^6^^–^^8^ phantom^9^^–^^11^ and *ex vivo* experiments,^12^^,^^13^ that this position is indifferent to scattering and that the absorption can be extracted and calculated. Therefore, it allows assessing absorption accurately, which is the main difference between blood and surrounding tissues in the visible-near infrared range.

## 2 Theory—Spatially Resolved Diffuse Reflectance on Endoscope Geometry

**Fig. 1:**
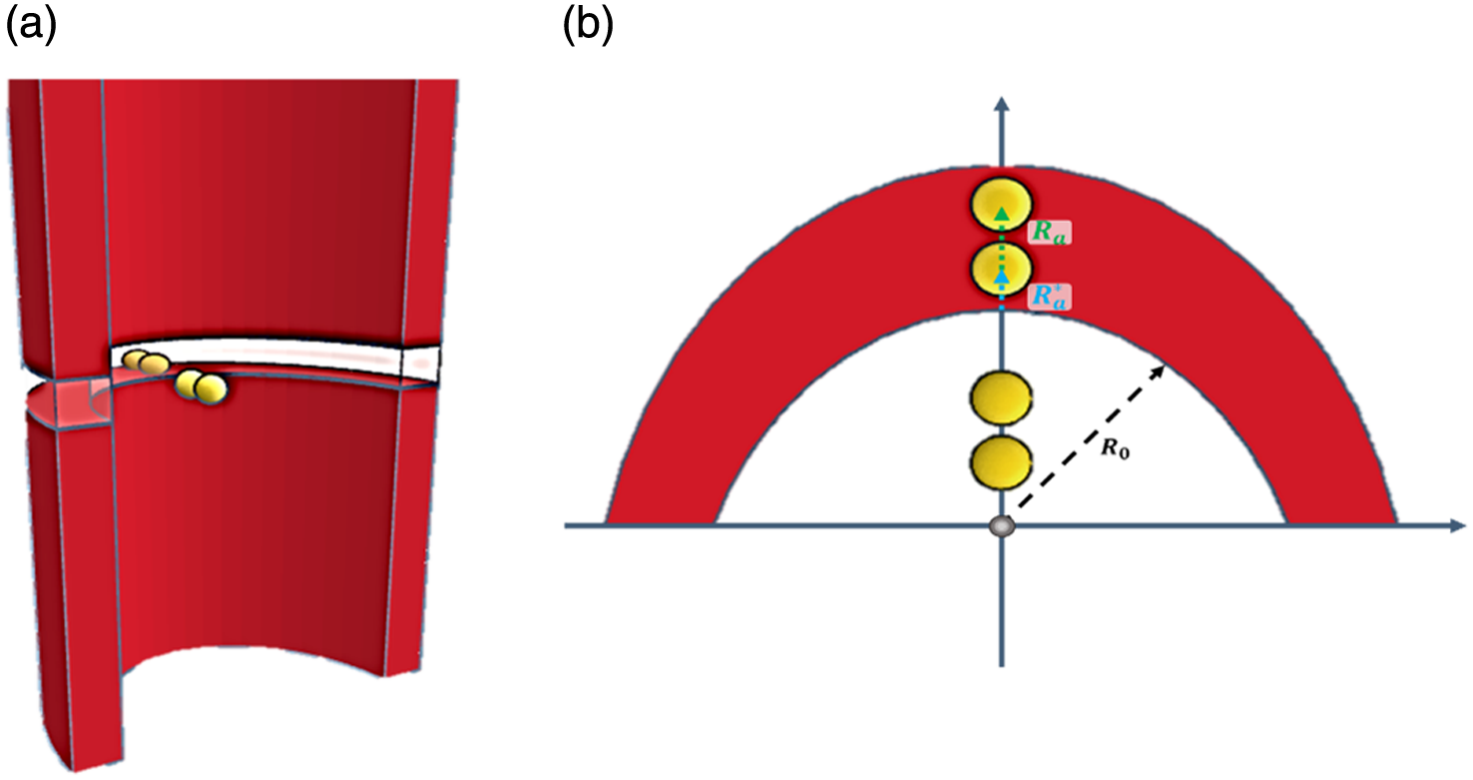
(a) Side view of the major and minor sources, inside and outside the tissue. (b) Superior view of the major and minor sources within the tissue cross-section area, both inside and outside the tissue, respectively.

In classical diffusion reflection (DR), the parallel light beam entering the tissue is replaced by a set of sources; one real source at a depth of $R_{\text{a}}={1/\mu }_{s}^{\prime }$ in the tissue, after the photons lose their original directionality, and a second image source to fulfill boundary conditions.^14^^,^^15^ However, this method does not fit in close distances to the light source. Previous work has demonstrated a simple approach to enhance the diffusion-based estimation of the DR at distances near the point-of-entry.^16^ As illustrated in Fig. 1, the approach suggested adding another set of sources to account for the shorter distances, the major source, and the minor source. Thus, two isotropic sources are defined in the tissue: a major real source at a distance $R_{\text{a}}$ from the tissue–air interface, and an additional minor source at a distance $R_{\text{a}}^{*}$ defined by

$$R_{\text{a}}^{*}=\frac{1-\eta }{R_{\text{a}}\mu_{\text{s}}^{2}},$$ (1)

where n=10 is an open parameter that affects the model’s sensitivity to the scattering anisotropy and was established in previous work.^14^ η is a slave-source index defined as

$$\eta ={\left[g\cdot \exp(1-g)\right]}^{\frac{1}{n}},$$ (2)

where g is tissue anisotropy. The intensity of the minor source S* is defined as

$$S^{*}=S\cdot \eta \cdot \exp(-\mu_{\mathrm{eff}}\frac{R_{\text{a}}+R_{\text{a}}^{*}}{2}).$$ (3)

This change enhances the match between analytical prediction and MC measurement of the IPL point.

**Fig. 2:**
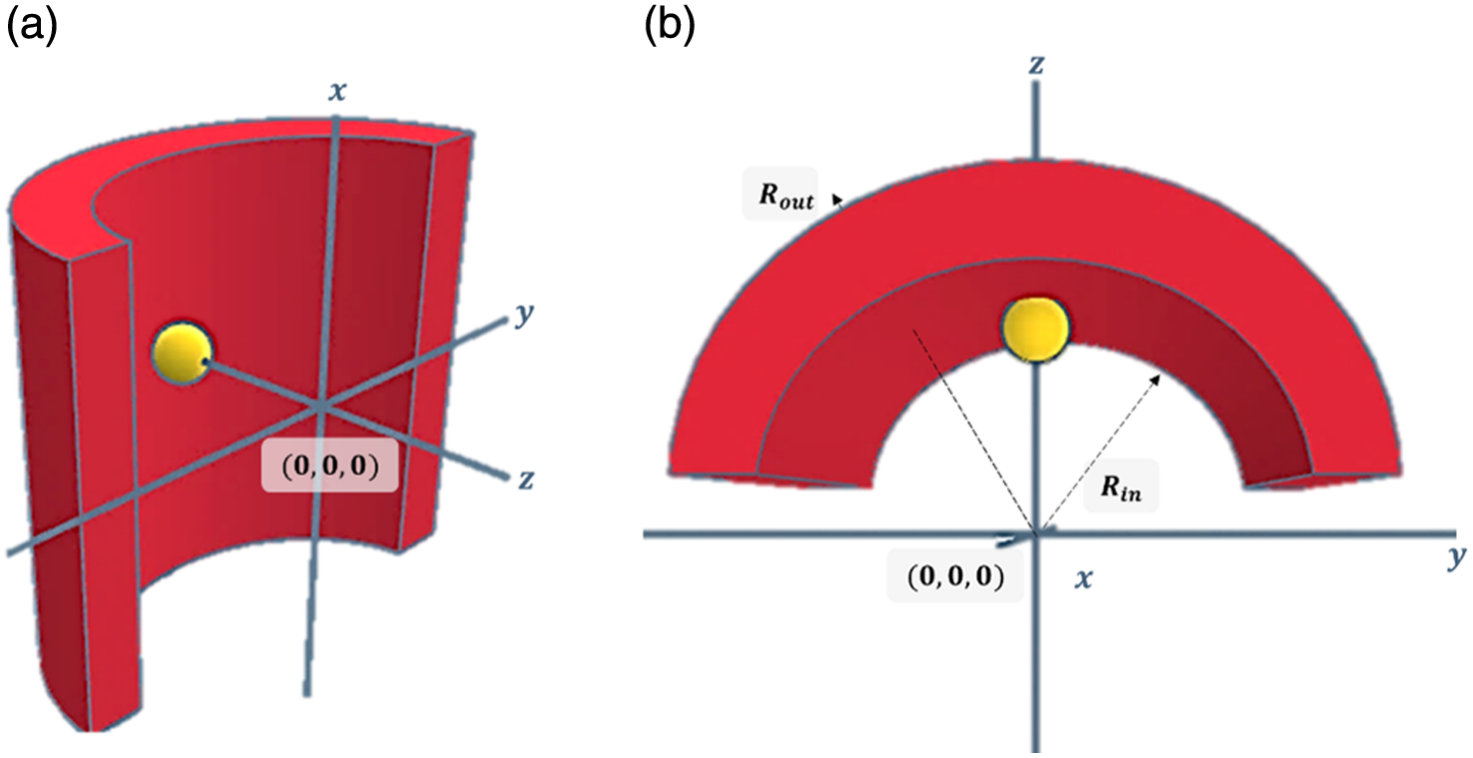
(a) Side view and (b) superior view of the simulation light source, positioned at the entrance of the cylindrical tissue.

We consider a convex tissue geometry of radius $R_{0}$ and infinite length. In cylindrical coordinates, the point-of-entry is denoted with $\left(R_{0},\varphi^{\prime },z^{\prime }\right)$, and the detector is denoted with $\left(R_{0},\varphi ,z\right)$. The line-of-sight distance between the point-of-entry and the detector is denoted by ρ. The diffusion coefficient D is defined as $D=1/(\mu_{a}+\mu_{s}^{\prime })$, and the effective attenuation coefficient is defined as $\mu_{\mathrm{eff}}=\sqrt{\mu_{\text{a}}/D}$. The major–minor dual-source configuration can thus be applied to convex geometry as in the semi-infinite geometry, by accounting for the curvature effect on the minor source as for the major source.

The photon fluence rate associated with the major source is described by^16^

$$\Psi (\rho ,\mu_{\text{a}},\mu_{\text{s}}^{\prime })=\frac{S}{4\pi D}[\frac{\exp(-\mu_{\mathrm{eff}}l_{\text{real}})}{l_{\text{real}}}-\frac{\exp(-\mu_{\mathrm{eff}}l_{\mathrm{imag}})}{l_{\mathrm{imag}}}\sqrt{\frac{R_{0}-z_{\text{a}}-2z_{\text{b}}}{R_{0}+z_{\text{a}}}}],$$ (4)

and the complementary minor source as

$$\Psi^{*}(\rho ,\mu_{\text{a}},\mu_{\text{s}}^{\prime })=\frac{S^{*}}{4\pi D}[\frac{\exp(-\mu_{\mathrm{eff}}l_{\text{real}}^{*})}{l_{\text{real}}^{*}}-\frac{\exp(-\mu_{\mathrm{eff}}l_{\mathrm{imag}}^{*})}{l_{\mathrm{imag}}^{*}}\sqrt{\frac{R_{0}-z_{\text{a}}^{*}-2z_{\text{b}}^{*}}{R_{0}+z_{\text{a}}^{*}}}].$$ (5)

We also evaluate two directions, one is along the longitudinal direction (where θ=180 deg), and the other one is along the azimuthal direction (where z=0).

In the longitudinal direction, the total DR of the endoscope geometry is the sum of the fluence rates of the major source and the minor source as follows:

$$R_{\mathrm{conC}}^{\mathrm{CW}}(z,\theta =180{}^{\circ},\mu_{\text{a}},\mu_{\text{s}},g)=\xi \frac{1}{4\sqrt{2}\pi }{\left[\Psi +\Psi^{*}\right]}^{\prime }$$ (6)

where ξ is a normalization factor for energy conservation, and

$$l_{\text{real}}(z,\theta =180{}^{\circ})=\sqrt{z^{2}+(z_{a}{)}^{2}}$$ (7)

$$l_{\text{image}}(z,\theta =180{}^{\circ})=\sqrt{z^{2}+(z_{a}+2z_{b}{)}^{2}},$$ (8)

$$l_{\text{real}}^{*}(z,\theta =180{}^{\circ})=\sqrt{z^{2}+(z_{a}^{*}{)}^{2}},$$ (9)

$$l_{\text{image}}^{*}(z,\theta =180{}^{\circ})=\sqrt{z^{2}+(z_{a}^{*}+2z_{b}^{*}{)}^{2}}.$$ (10)

Similarly, along the azimuthal direction:

$$R_{\mathrm{conC}}^{\mathrm{CW}}(z=0,\theta ,\mu_{\text{a}},\mu_{\text{s}},g)=\xi \frac{1}{4\sqrt{2}\pi }[\Psi +\Psi^{*}],$$ (11)

where in this case:

$$l_{\text{real}}(z=0,\theta )=\rho \sqrt{1+\frac{(z_{a}{)}^{2}}{\rho^{2}}+\frac{z_{a}}{R_{0}}},$$ (12)

$$l_{\text{image}}(z=0,\theta )=\rho \sqrt{1+\frac{(z_{a}+2z_{b}{)}^{2}}{\rho^{2}}-\frac{z_{a}+2z_{b}}{R_{0}}},$$ (13)

$$l_{\text{real}}^{*}(z=0,\theta )=\rho \sqrt{1+\frac{(z_{a}^{*}{)}^{2}}{\rho^{2}}+\frac{z_{a}^{*}}{R_{0}}},$$ (14)

$$l_{\text{image}}^{*}(z=0,\theta )=\rho \sqrt{1+\frac{(z_{a}^{*}+2z_{b}^{*}{)}^{2}}{\rho^{2}}-\frac{z_{a}^{*}+2z_{b}^{*}}{R_{0}}},$$ (15)

when the line-of-sight distance ρ found according to the angle (in degree) by

$$\rho =2R_{0}\;\sin(\frac{180-\theta }{2})$$ (16)

## 3 Methods and Materials

### 3.1 Monte Carlo Simulation

Monte Carlo (MC) simulations of a cylindrical air hole surrounded by scattering tissue were developed to study the influence of the scattering on the backscattered intensity. Our MC simulation defines the air–tissue interface by the equation of a circle, and relies on light transport in the tissue model of OMLC.^17^ As shown in Fig. 2, the medium is characterized by the cylinder tissue with an internal radius $R_{\text{in}}$, external radius $R_{\text{out}}$, reduced scattering coefficient $\mu_{\text{s}}^{\prime }$ and anisotropy factor g. All photons enter the medium at a single isotropic point ($z=R_{\text{in}}$, x=0, y=0), and with the same parallel direction ($u_{x}=0$, $u_{y}=0$, $u_{z}=1$). The step size $p_{s}$ between successive interaction events was randomly sampled from an exponential distribution:

$$p_{\text{s}}=-\frac{\log(\text{rand})}{\mu_{\text{t}}},$$ (17)

where rand is a random number drawn from a uniform distribution between 0 and 1 (rand∼U(0,1)), and $\mu_{\text{t}}=\mu_{\text{a}}+\mu_{\text{s}}$ is the total interaction coefficient.

If the photon was scattered, the new photon trajectory was determined according to the Henyey–Greenstein phase function,^18^ where the cosine of the scattering angle was sampled using:

$$\cos(\theta )=\frac{1+g^{2}-(\frac{1-g^{2}}{1-g+2g\cdot \text{rand}})}{2g},$$ (18)

in either the polar or vertical direction. Once the photon reencounters the air–tissue interface, it is counted, and its position is saved. In this study, we neglected the absorption since we are only interested in proving the existence of two IPL points: the azimuthal and the longitudinal ones.

### 3.2 Phantom Preparation

Phantoms are synthetic tissues used for mimicking the optical properties of almost any organ.^19^^,^^20^ Calibrating optical instruments on actual tissue is usually tough to perform and is very hard to analyze. Phantom samples give the ability to mimic organs with any optical property in every desired shape, which allows us to perform experiments in a controlled way with known material optical properties. In general, there are two types of phantoms: liquid phantoms and solid phantoms; and the use of those two types depends on the sample we want to mimic. For this project, we designed three different polydimethylsiloxane (PDMS) hollow cylinders solid phantom with different scatterings, and one hemoglobin-agar based phantom, to imitate the human esophagus.

For the first experiment, we prepared the 3 PDMS (SYLGARD™ 184 Silicone Elastomer Kit, Dow Corning, United States) based phantoms with the same air hole diameter of 1 cm and different reduced scattering coefficients ($\mu_{\text{s}}^{\prime }=16$, 18, $26\;{\mathrm{cm}}^{-1}$ scattering coefficient at a red wavelength ∼632.8  nm). The material used to introduce the scattering properties was ${\mathrm{TIO}}_{2}$ nanoparticles (NO-0051-HP, Ionic Liquids Technologies, GmbH, Germany), and by varying their concentration, the phantoms with different scattering profiles were designed. In the first stage of preparation, using a sonication bath, the PDMS activator was mixed with the appropriate amount of the ${\mathrm{TIO}}_{2}$ nanoparticles in a sealing plastic jar. Once the ${\mathrm{TIO}}_{2}$ and the activator were well mixed, the PDMS base was added to the activator-${\mathrm{TIO}}_{2}$ solution and mixed again. After getting a uniform texture, the solution was poured into a thermal glass mold and placed in the vacuum chamber for a degassing process. As the PDMS-${\mathrm{TIO}}_{2}$ is very thick, it is necessary to repeat the degassing process a couple of times, until getting a smooth solution, without any air bubbles on the surface. Finally, for solidification, the solution must rest at room temperature for 48 h. After 48 h of curing, the samples were extracted from the mold gently without causing any damage. Because PDMS samples are very flexible, it is impossible to drill them into smooth hollow cylinders. Therefore, the samples were frozen in liquid nitrogen to harden them before drilling. Once the samples were hard enough, they were fixed on the PNC drilling with a 1 cm HSS and were drilled to get the desired cylindrical structure.

**Fig. 3:**
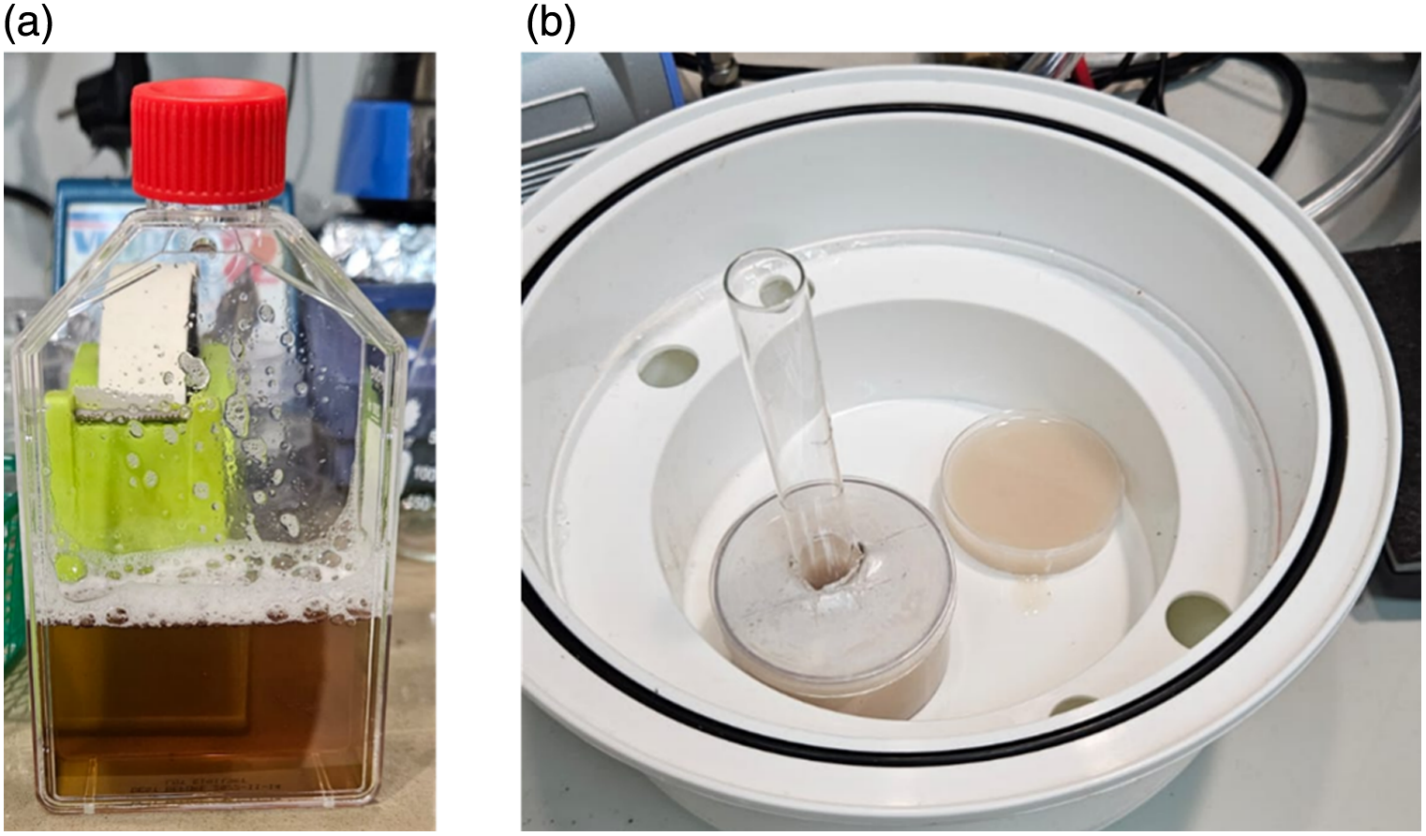
(a) Hemoglobin–water mixture, (b) two hemoglobin phantoms placed in the vacuum chamber, before the degassing process, one in a simple petri plate for controls, and the other as a hollow cylindrical tissue sample for the experimental setup.

Then, for the next experiment, an agar phantom mixed with hemoglobin (NO-H7379 Sigma-Aldrich, St. Louis, Missouri, United States) and Intralipid (NO-I141 Sigma-Aldrich) was prepared. This phantom is used as a controlled sample for calculating the different optical properties needed such as the differential pathlength factor (DPF).^12^ First, 2 g of agar (NO-A9045 Sigma-Aldrich) was mixed with double-distilled water and heated in a thermoformed glass mold for 3 h, until reaching a uniform texture. Parallelly, the hemoglobin was dissolved with double-distilled water in a plastic tube, as shown in Fig. 3(a), and measured in a spectrophotometer (UV – 1900, Shimadzu, Japan), to ensure an absorption coefficient of $\mu_{\text{a}}=0.25\;{\mathrm{cm}}^{-1}$ at 633 nm. Next, the Intralipid was mixed with distilled water in a separate tube. Once the Agar-water mixtures were well mixed, the Intralipid-water and the hemoglobin-water were added and mixed for 1 min. Once the mixing process finished, the mixture was poured into a small glass dish with a 1.6 cm diameter tube fixed in the middle, to create an air cylindrical hole in the phantom. Then, the phantom was placed into a vacuum chamber to avoid air bubbles for 24 h, until it became completely solid, as shown in Fig. 3(b). In the end, for the second experiment, there was one PDMS phantom obtained from the first experiment used as a nonabsorbing tissue, and one hemoglobin-agar phantom used as a control sample with a known absorption coefficient.

Finally, for the *ex vivo* sample, a thick piece of chicken breast tissue was carefully wrapped around a glass tube with the muscle fibers parallel to the vertical axis, thereby forming a uniform hollow cylindrical tissue model.

### 3.3 Experimental Setup

**Fig. 4:**
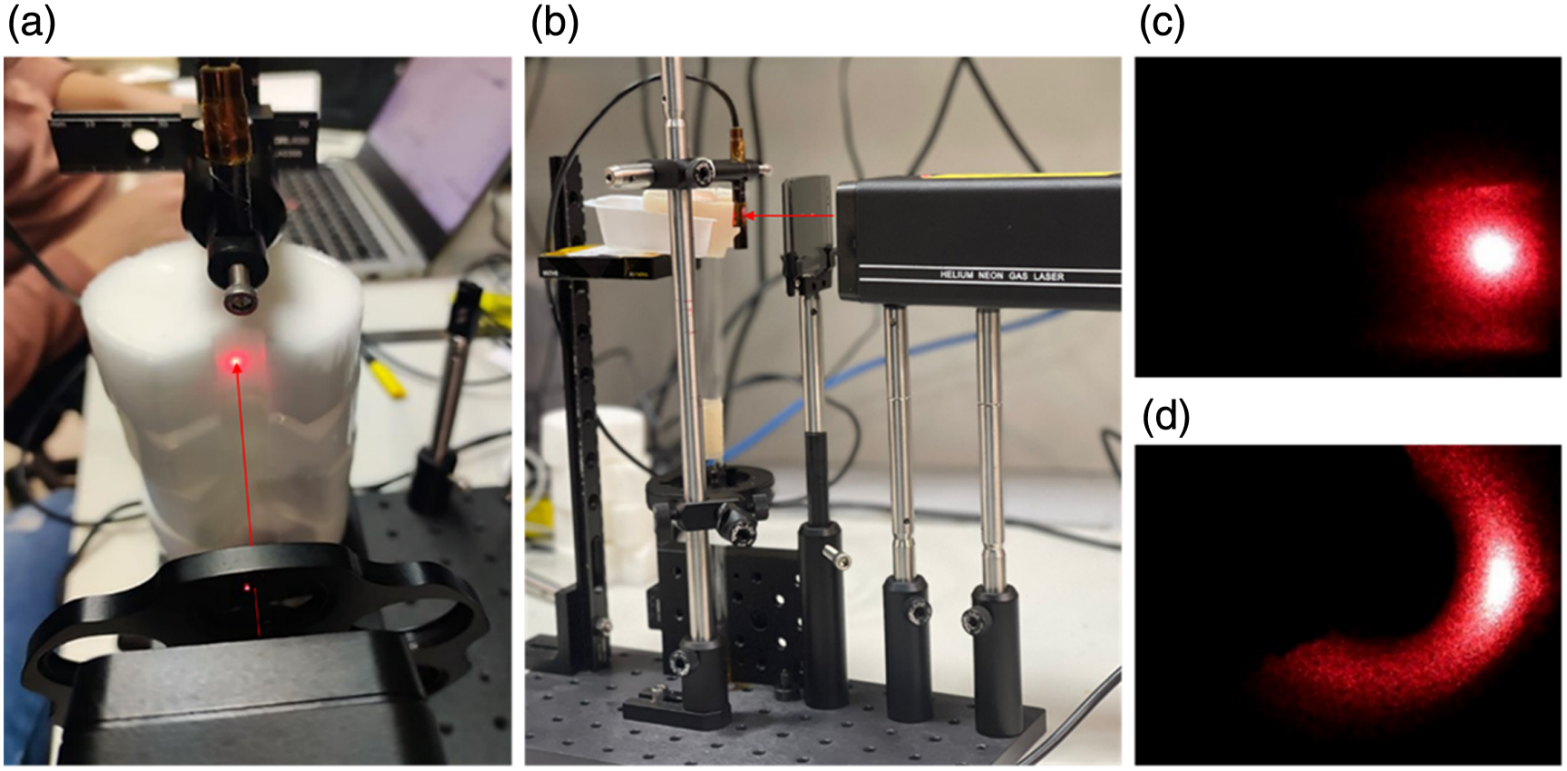
(a) Endoscope-based setup—three phantoms stacked vertically, illuminated by a red laser directed along the red arrow and photographed using the endoscope equipped with the removable mirror. (b) Side view of the second setup for the *ex vivo* measurements: a hemoglobin-based phantom placed on the stand, illuminated by the laser and photographed by the endoscope. (c) Full scattering profile captures from the azimuthal measurements, (d) the longitudinal captures from the vertical measurements.

To find the IPL point, we build an experimental setup which allows us to collect data from a phantom in the form of surface reflection, which can be provided by a regular endoscope picture (Fig. 4). In this experiment, a USB endoscope camera was used rather than a clinical endoscope; therefore, a mirror was added to rotate the point of view by 90 deg. For our optical system, a helium-neon gas laser was used with a wavelength of 632.8 nm and 0.8 mW power (HNLS008L, Thorlabs, Japan), an optical attenuator, and an endoscope with a removable angular mirror. In addition, the phantoms were placed on a manual lift platform and one on top of each other to obtain a uniform measurement distance for all the samples. In the longitudinal part measurements, the endoscope together with the angular mirror was placed toward the laser–phantom interaction spot in a fixed place, whereas the samples were changed between frames using the manual lift platform. At first, we aligned the picture by rotating the endoscope to obtain pictures that aligned with the cylinder’s longitudinal axis, and simplify the extraction of the results. After ensuring that the stage of the platform is straight by using a lever, a ruler was placed on the air-phantom interface to empirically assess the ground sample distance (GSD) of the camera, at the given distance. In the azimuthal part measurements, the angular mirror was removed, and the endoscope was placed on top of the cylindrical hole while both the phantoms and laser remained in the same place as before.

In the second experiment, a cylindrical glass tube was fixed onto the platform, and the phantoms were wrapped around the back of the tube. Each phantom was measured in the same manner as described previously, including the Agar-hemoglobin phantom. For the final *ex vivo* measurements, a chicken breast of 2 cm thickness was wrapped around the tube and measured. It is important to note that all calibration procedures were performed as in the first experiment, i.e., placing the ruler at the sample–laser interaction point and extracting the GSD of the camera at that distance. Another important note is that the supplement of the glass tube into the system does not affect the results since the exact input intensity is not required, and any external reflections were directed outside the camera’s field of view.

### 3.4 Image Processing

**Fig. 5:**
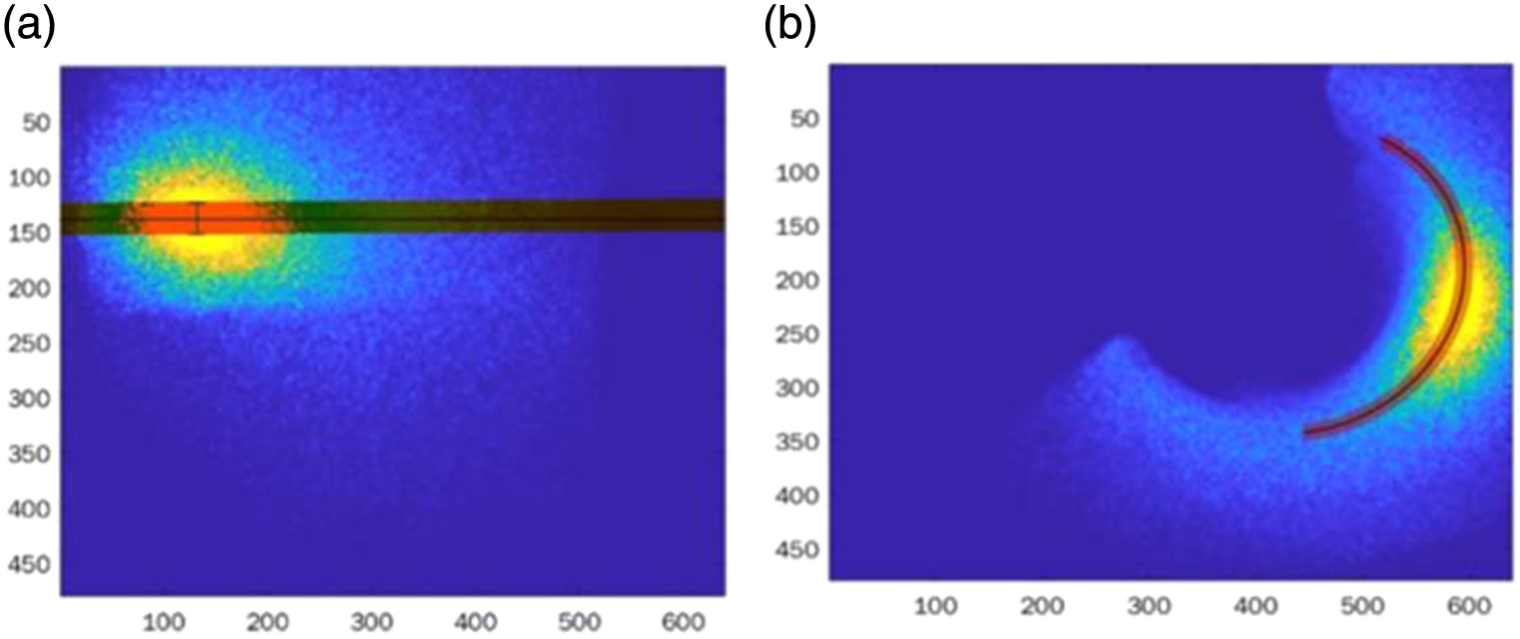
Image processing of (a) longitudinal and (b) azimuthal measurements.

To extract the diffusion profile as a function of distance and angle, proper calibration and image processing were required. The alignment described above (Sec. 3.3) simplified the analysis of the longitudinal measurements [Fig. 5(a)]. First, we found the place with maximal power and defined it as the beam center. Then, we averaged several rows [red area marked on Fig. 5(a)] together to improve the signal to noise ratio (SNR) of the measurements.

For the azimuthal measurements, we first found the center for each picture individually. Then, we defined the polar coordinates according to these centers and defined the radius with the maximal average value as the desired circumference. Next, we averaged a ring area [the red area marked on Fig. 5(b)] and divided it into equal slices. The angle with the maximal value was defined as the center of the beam (180 deg in our notation).

## 4 Results

### 4.1 Theory—Spatially Resolved Diffuse Reflectance on Endoscope Geometry

**Fig. 6:**
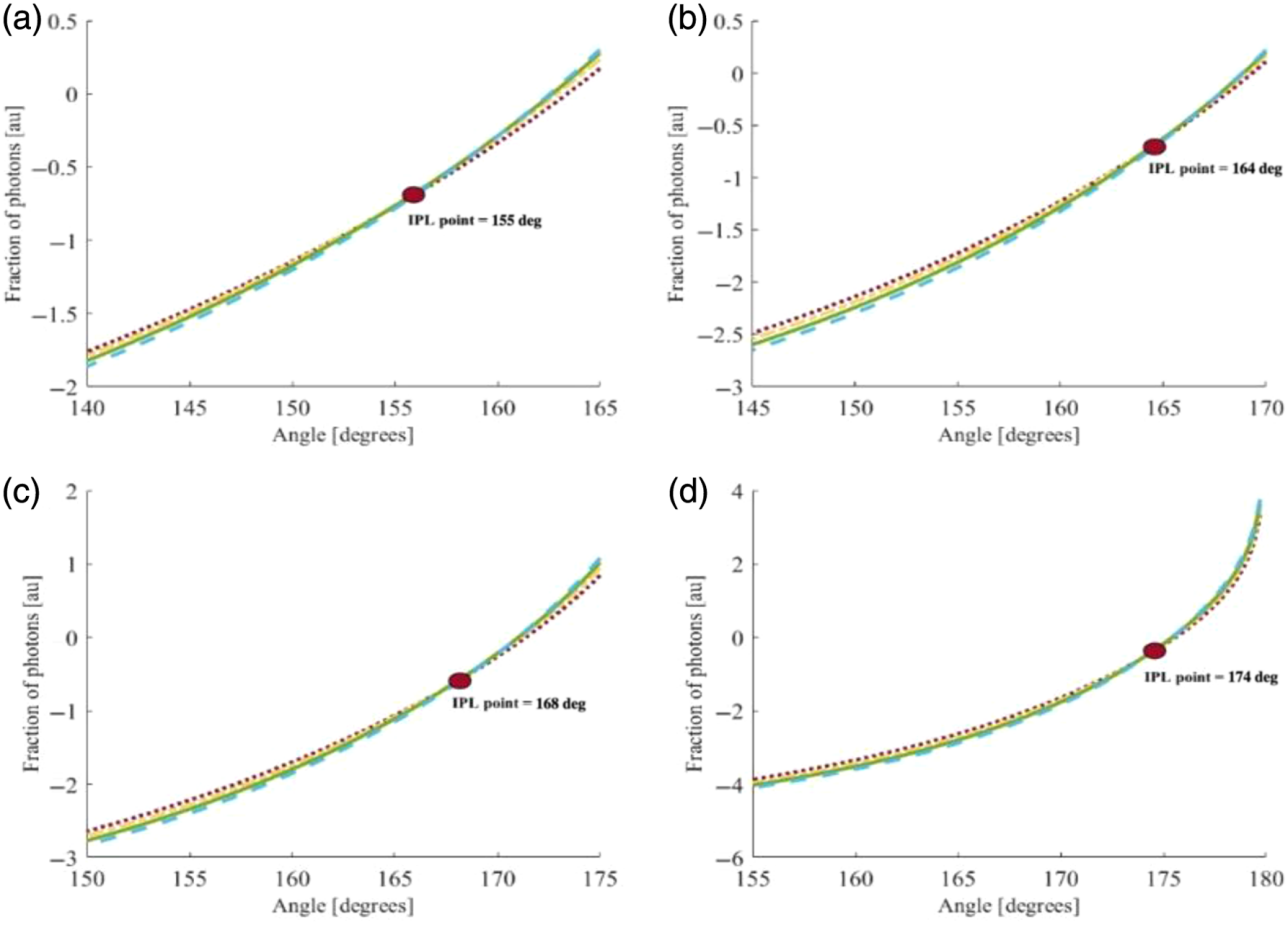
IPL point evaluated along the azimuthal direction for convex cylindrical tissues with different reduced scattering coefficients ($\mu_{\text{s}}^{\prime }=22$, 26, 30, and $34\;{\mathrm{cm}}^{-1}$, represented by blue, green, yellow, and red, respectively) and with radii of 0.5 cm (a), 0.8 cm (b), 1.0 cm (c), and 2.0 cm (d). The theoretical model predicts that the IPL point appears at different azimuthal angles depending on the tissue radius.

**Fig. 7:**
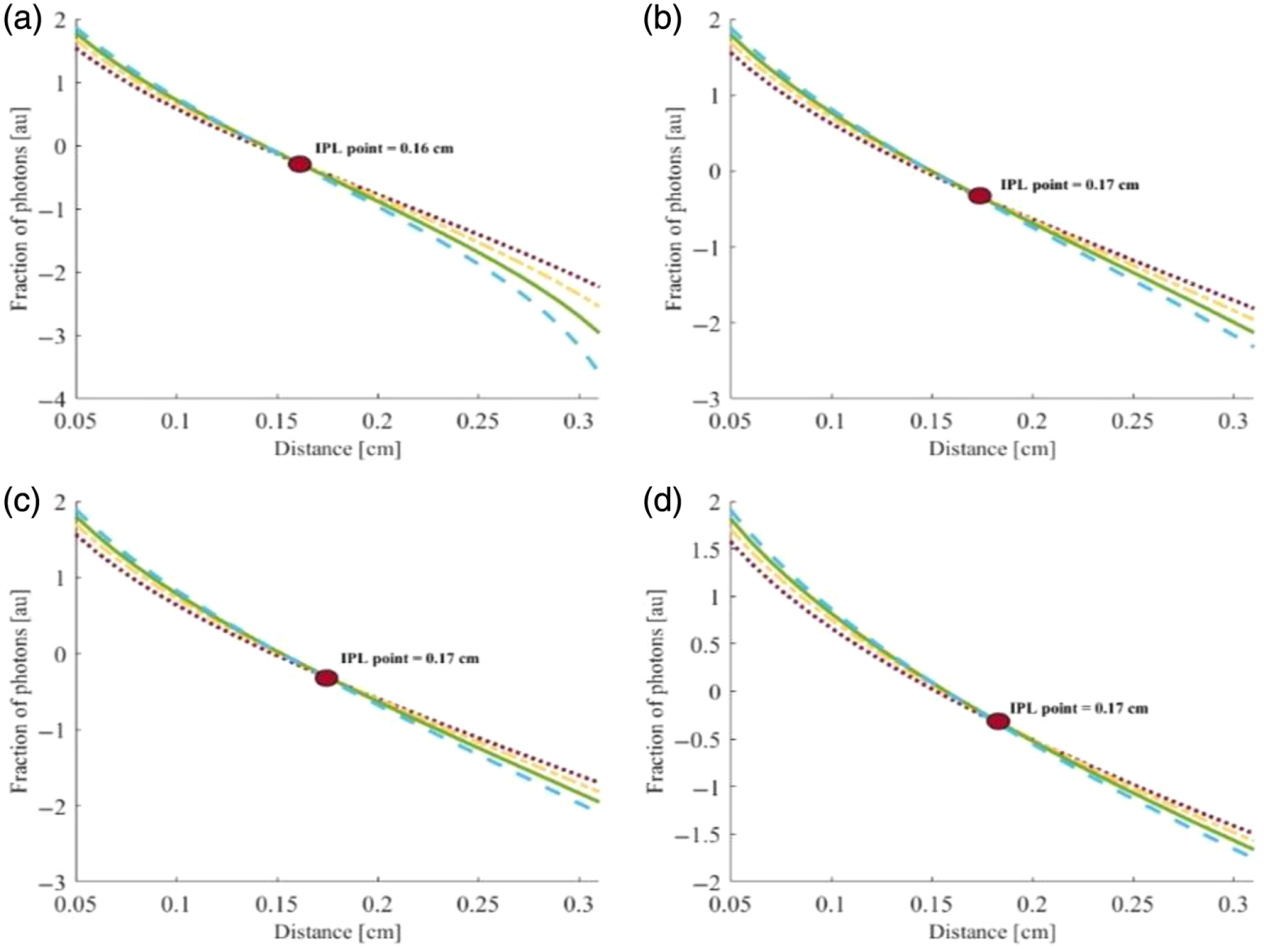
IPL point evaluated along the vertical direction for convex cylindrical tissues with different reduced scattering coefficients ($\mu_{\text{s}}^{\prime }=22$, 26, 30, and $34\;{\mathrm{cm}}^{-1}$, represented by blue, green, yellow, and red, respectively) and with radii of 0.5 cm (a), 0.8 cm (b), 1.0 cm (c), and 2.0 cm (d). The theoretical model predicts that the IPL point appears at about the same place independent of the sample radius.

First, a calculation of the spatially resolved diffuse reflectance based on four different reduced scattering coefficients ($\mu_{\text{s}}^{\prime }=22$, 26, 30, and $34\;{\mathrm{cm}}^{-1}$, represented by blue, green, yellow, and red, respectively) was performed in both the azimuthal and vertical directions, as described in Sec. 2. The analysis was conducted for four different radii of the cylindrical tissue (R=0.5, 0.8, 1, 2 cm). The resulting full scattering profiles (FSPs) corresponding to the various reduced scattering coefficients are presented in Figs. 6 and 7, respectively.

For the azimuthal direction, the curves that initially show the highest intensity values at the origin appear lowest at the center of the light (θ=180 deg), and vice versa. This inversion creates a distinct intersection point common to all curves, representing the IPL point. Furthermore, the results show that as the sample radius increases, the position of the IPL point also shifts outward, as previously demonstrated.^7^

As for the azimuthal direction, in the vertical direction, the highest intensity values at the origin appear lowest at the points farthest from the laser center (D>0.2  cm), which creates the inversion between the curves and represents the IPL. In contrast to the azimuthal results, in this case, the IPL point remains constant, independent of the sample radius, which is consistent with the theoretical prediction for a half-infinite geometry.^6^

### 4.2 MC Simulation of the IPL Point from Endoscopy Geometries of a Cylindrical Air Hole

**Fig. 8:**
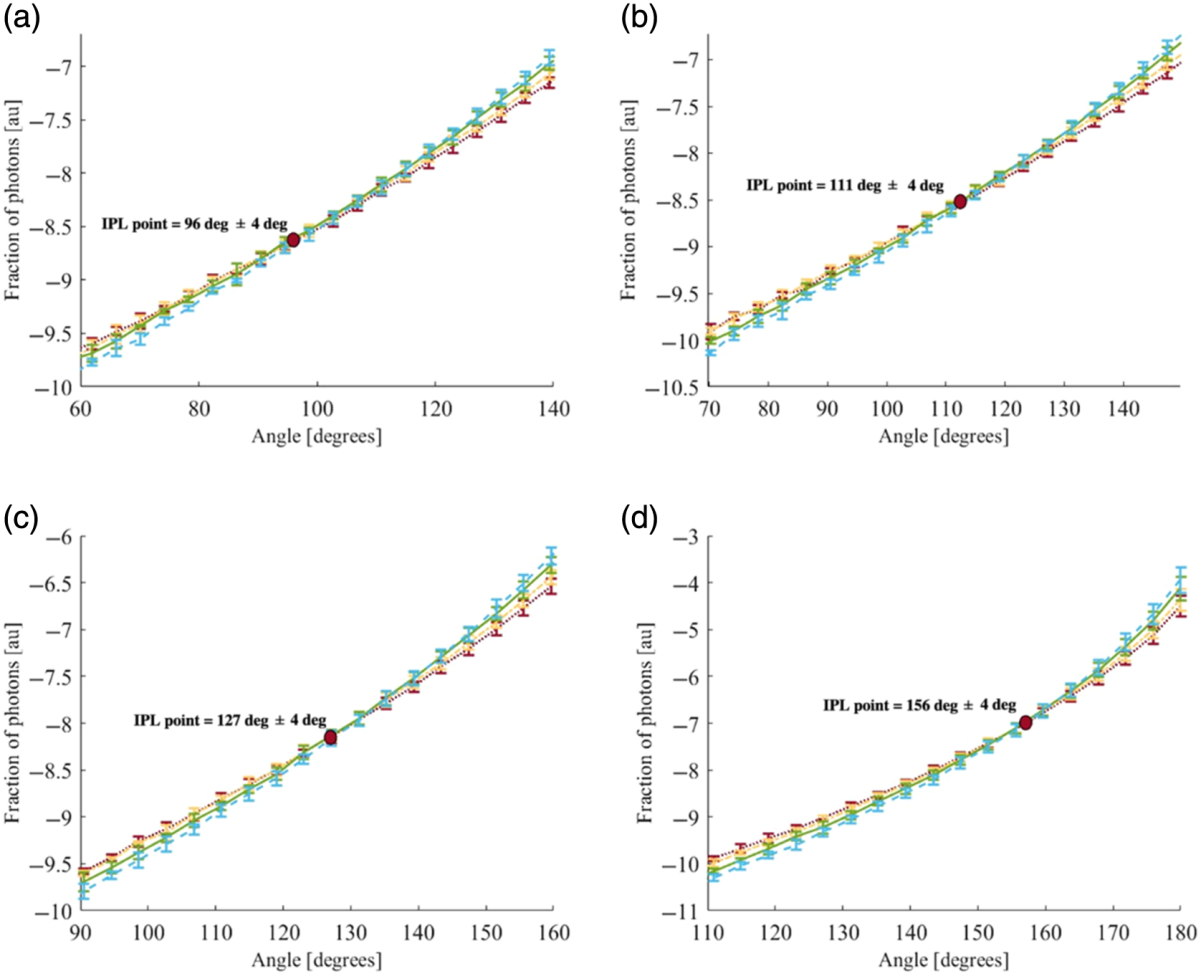
IPL evaluated along the azimuthal direction for a cylindrical air hole with radii of 0.5 cm (a), 0.8 cm (b), 1.0 cm (c), and 2.0 cm (d), and for different reduced scattering coefficients ($\mu_{\text{s}}^{\prime }=16$, 18, 22, and $26\;{\mathrm{cm}}^{-1}$, represented by blue, green, yellow, and red, respectively). The MC simulations predict that the IPL appears at various azimuthal angles, depending on the radius.

**Fig. 9:**
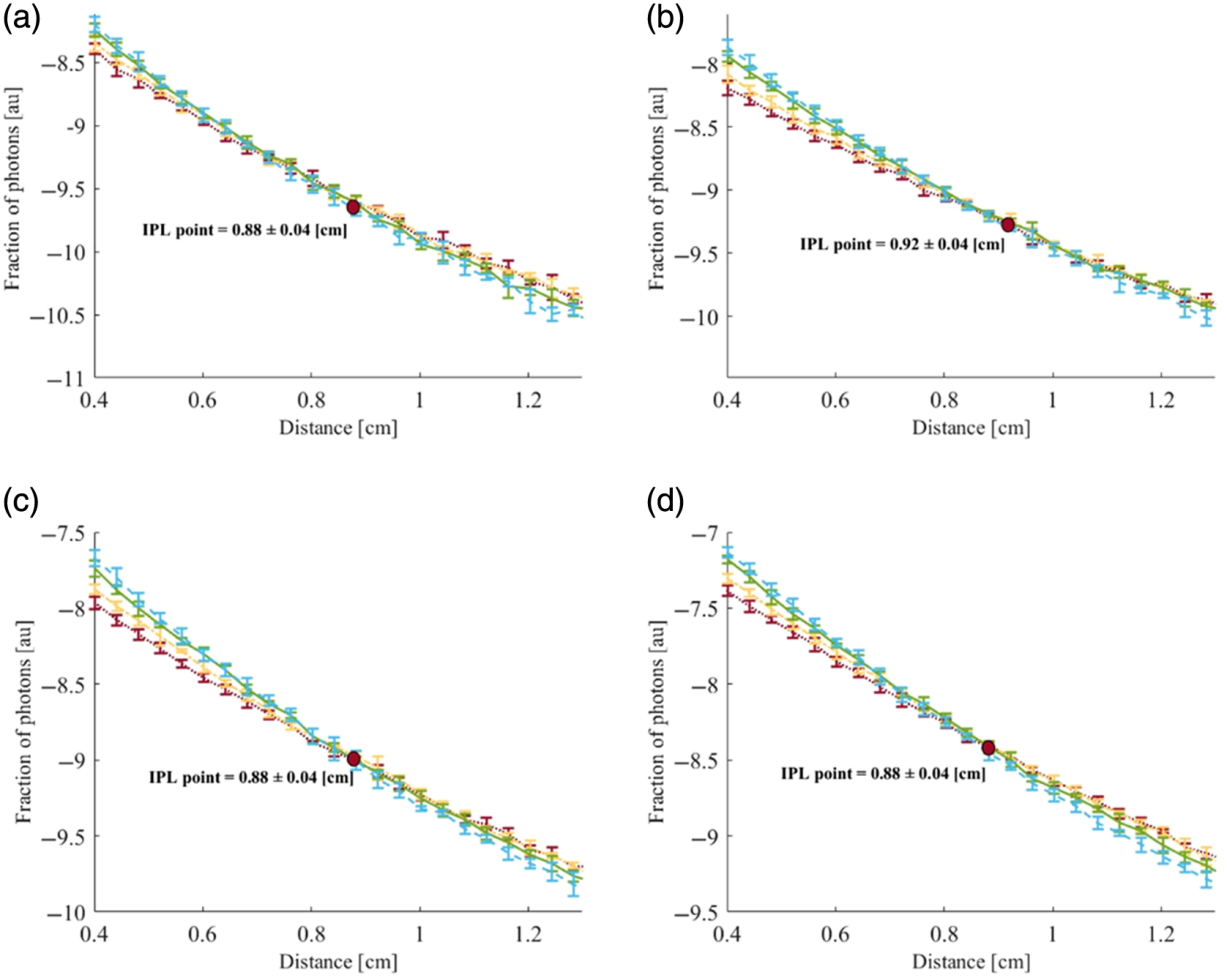
IPL evaluated along the vertical direction for a cylindrical air hole with radii of 0.5 cm (a), 0.8 cm (b), 1.0 cm (c), and 2.0 cm (d), and for different reduced scattering coefficients ($\mu_{\text{s}}^{\prime }=16$, 18, 22, and $26\;{\mathrm{cm}}^{-1}$, represented by blue, green, yellow, and red, respectively). The MC simulations predict the IPL point consistently within a well-defined region.

Second, a simulation of four different scattering profiles ($\mu_{\text{s}}^{\prime }=16$, 18, 22, and $26\;{\mathrm{cm}}^{-1}$, shown in alternating shades of blue, green, yellow, and red, respectively, in Figs. 8 and 9, and compatible with the designed phantoms) and the four different radii as in the theory (R=0.5, 0.8, 1, 2 [cm]), were performed in both the azimuthal and vertical directions, described in Sec. 3.1—Monte Carlo simulation. The FSPs corresponding to the reduced scattering coefficients are presented in Figs. 8 and 9.

Consistent with the theoretical prediction, the azimuthal IPL point shifts to higher values as the sample radius increases. In this case as well, the IPL is formed at the intersection of the profiles.

These findings, shown in Fig. 9, further confirm that the vertical IPL values across all radii are nearly the same, in full agreement with the theoretical model. It should be noted that the exact IPL locations differ between the theory and the simulations due to inherent discrepancies between the two approaches. Nevertheless, both exhibit the same overall behavior.

### 4.3 Experimental Results of Endoscopy Geometries

Next, we prepared the phantoms with different scatterings, as described in Sec. 3.2, and measured them according to the description in Sec. 3.3. The pictures from the endoscope were analyzed according to Sec. 3.4.

**Fig. 10:**
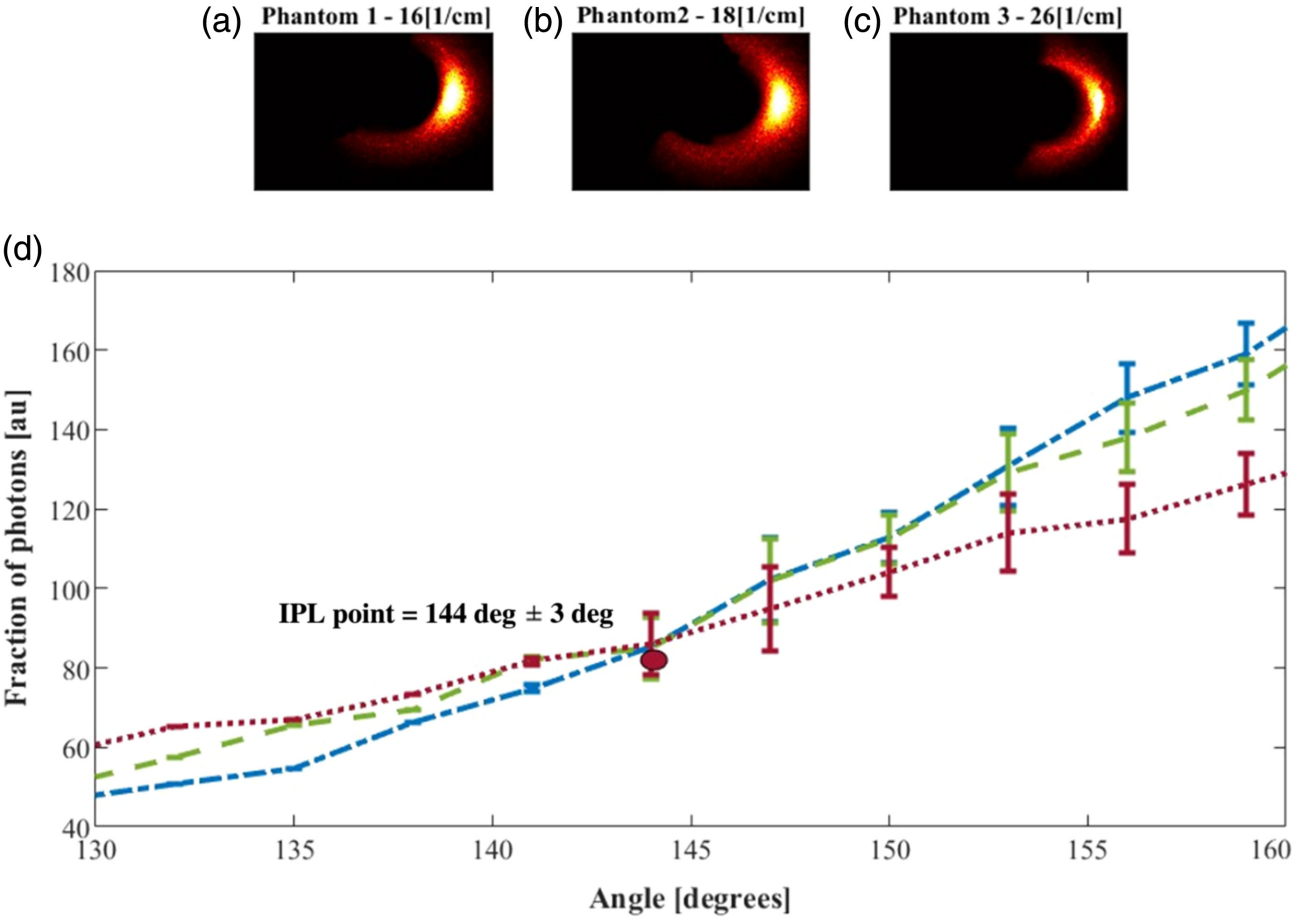
FSP measurements along the azimuthal direction of a cylindrical air hole radius of 0.5 cm. The endoscopy pictures of phantoms with a reduced scattering coefficient of (a) 16, (b) 18, (c) $26\;{\mathrm{cm}}^{-1}$. (d) The FSPs of these phantoms have an IPL point at 144±3 deg.

The full scattering profile (FSP) measured in the azimuthal direction, corresponding to phantoms with $\mu_{\text{s}}^{\prime }=16$, 18, and $26\;{\mathrm{cm}}^{-1}$, were analyzed over a ring-shaped region [Fig. 10(a)–10(c)], The FSPs reveal an IPL point at an angle of 144  deg±3  deg [Fig. 10(d)].

**Fig. 11:**
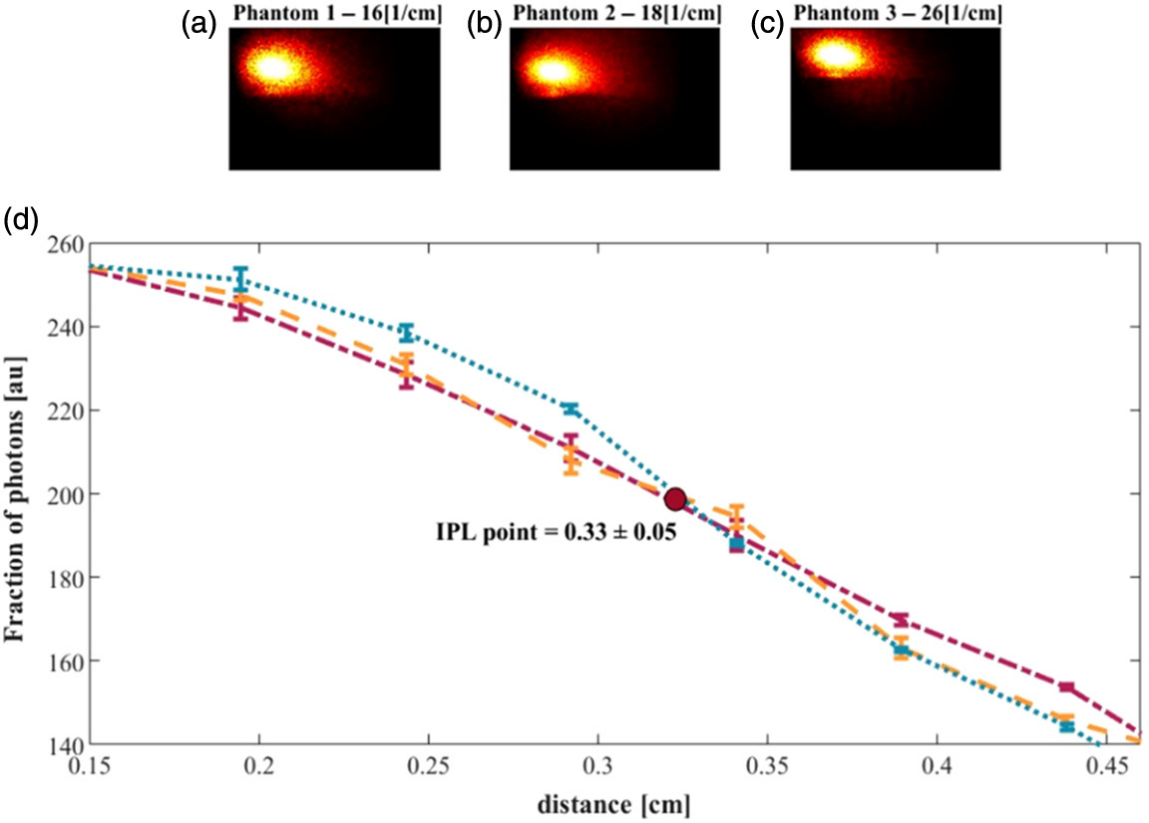
Reflectance measurements along the vertical direction of a cylindrical air hole radius of 0.5 cm. The light reflectance of phantoms, with a reduced scattering coefficient of (a) 16, (b) 18, (c) $26\;{\mathrm{cm}}^{-1}$, were extracted from endoscopy pictures. An IPL point is apparent at 0.33±0.05  cm.

Similarly, the same phantoms were measured in the vertical direction and analyzed from a rectangular area [Fig. 11(a)–11(c)]. The DR intensity profiles [Fig. 11(d)] present an IPL point at about 0.33±0.05  cm.

In contrast to the theoretical and simulation results, the experimental data do not exhibit a clearly defined intersection point. This discrepancy is likely due to various environmental variations affecting the measurements. Nevertheless, a distinct inversion between the curves is observed within a specific region, which represents the location of the IPL point.

### 4.4 *Ex Vivo* Experimental Results of Endoscopy Geometries

**Fig. 12:**
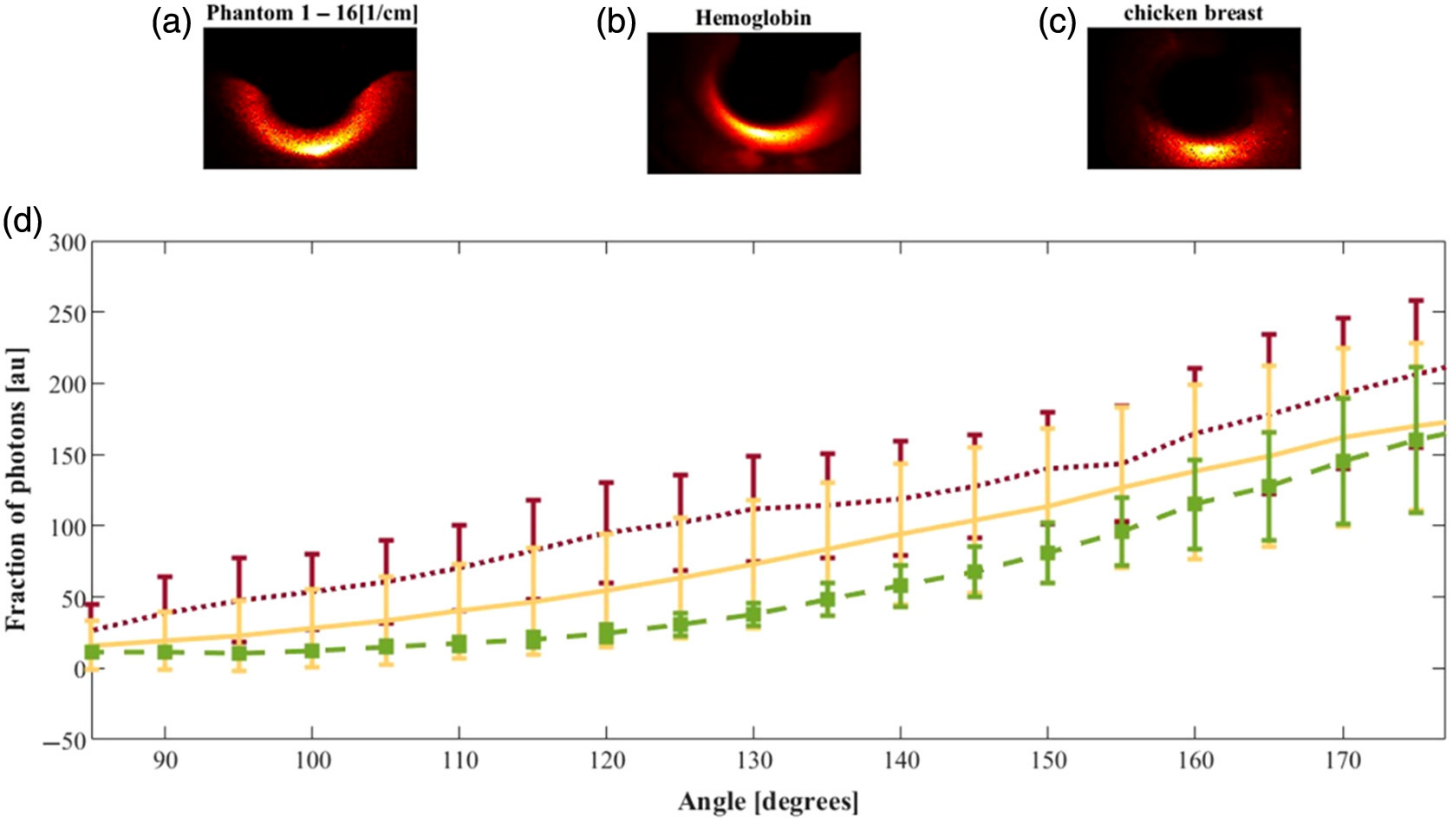
FSP measurements along the azimuthal direction of a cylindrical air hole with a radius of 0.8 cm. The FSPs of the phantom with a reduced scattering coefficient of (a) $16\;{\mathrm{cm}}^{-1}$, (b) the hemoglobin phantom, and (c) the chicken breast are (d) marked in red, yellow, and green, respectively.

A PDMS phantom with the hemoglobin mentioned above was measured one after the other, centered around a glass tube. Then, the chicken breast enveloping the same glass tube was placed on the platform and measured. Figure 12 shows the intensity reflected from the three tissues as a function of the azimuthal axis, to find the *ex vivo* absorption properties.

To calculate the absorption coefficient of the chicken, it is necessary to find the ${\mathrm{DPF}}_{0}$, which can be inferred from the hemoglobin phantom using the knowledge of its absorption at 633 nm measured in Sec. 3.2.^12^ Based on the IPL point found in Sec. 4.3, and given that the PDMS phantoms present negligible absorption, the DPF in the case of no absorption, i.e., ${\mathrm{DPF}}_{0}$, is calculated according to the following equation, which describes the relationship between the ${\mathrm{DPF}}_{0}$, the IPL point, and the absorption coefficient of the sample:

$${\mathrm{DPF}}_{0}=-\frac{1}{\mu_{\text{a}}\sqrt{R}\cdot D\cdot \sin(\frac{180-\theta_{\mathrm{IPL}}}{2})}\;\ln(R);\;R=\frac{I_{t}(\theta_{\mathrm{IPL}})}{I_{0}(\theta_{\mathrm{IPL}})},$$ (19)

where D is the diameter of the sample, and R is the ratio between the intensity of the hemoglobin sample and the phantom. The nominal ${\mathrm{DPF}}_{0}$ obtain from the results is: ${\mathrm{DPF}}_{0}=3.44$ within the range of $1.81\leq {\mathrm{DPF}}_{0}\leq 12.6$, respectively to the results standard deviation (STD). It is important to notice that each sample was measured individually, i.e., the ratio R is not affected by the difference between the refractive indices of the samples.

Finally, by resolving the equation with the calculated ${\mathrm{DPF}}_{0}$, it is possible to get the absorption of the chicken breast. This time, based on the phantom’s intensity and the intensity reflected from the chicken tissue, the calculated nominal absorption is: $\mu_{\text{a}}=0.94\;{\mathrm{cm}}^{-1}$, within the range of $0.2\;{\mathrm{cm}}^{-1}\leq \mu_{\text{a}}\leq 2\;{\mathrm{cm}}^{-1}$, which matches known literature values.^21^^,^^22^

## 5 Discussion and Conclusions

In this work, we experimentally validated the existence of a self-calibration phenomenon, namely, the IPL point, in an endoscopy scenario. Previously, this phenomenon was demonstrated in concave cylindrical and half-infinite geometries, both numerically (via MC simulations^6^^–^^8^ and diffusion theory^23^) and experimentally (with phantoms,^9^^–^^11^ *ex vivo* tissues^12^ and *in vivo*^13^). We also suggested that an IPL point should exist in endoscopic geometry, specifically in a cylindrical air hole surrounded by tissue.^6^ Here, we presented and validated the diffuse theory for convex geometry, confirmed this theory using MC simulations, and finally, for the first time, demonstrated it experimentally. We also show how to use this phenomenon to extract absorption from turbid media.

The diffusion theory results showed the existence of an IPL point for all radii taken in highly scattering tissues, consistent with the MC simulations and phantom experiments. However, in low-scattering tissues, the theoretical assumptions break down as the short source–detector separations employed in Sec. 4.1 yield insufficient scattering. Moreover, as presented in Fig. 7, the IPL point in the longitudinal direction does not change as a function of the radius, which supports the previous theory for half-infinity surfaces.^6^

Both MC simulations and diffusion theory predict two IPL points: one along the azimuthal axis and one along the longitudinal axis, each one corresponding to its absorption coefficient. Importantly, the IPL locations in both directions should be consistent, i.e., the arc length from the azimuthal results ($L_{\mathrm{IPL}}$) should be equal to the longitudinal IPL distance ($D_{\mathrm{IPL}}$). Using the relation^12^

$$L_{\mathrm{IPL}}=D\cdot \sin(\frac{180-\theta_{\mathrm{IPL}}}{2}),$$ (20)

where D is the sample diameter and $\theta_{\mathrm{IPL}}$ is the angle at which the IPL occurs. We compared all the vertical and azimuthal results from the theory calculation and MC simulations (Table 1).

**Table 1:** Validation between the azimuthal and the vertical results, both theory and simulation.

Sample radius	Arc distance (theory) [cm]	Vertical distance (theory) [cm]	Arc distance (MC) [cm]	Vertical distance (MC) [cm]
(a) : R=0.5 cm	$L_{\theta =155}=0.20$	$D_{\mathrm{IPL}}=0.16$	$0.63\leq L_{\theta =98}\leq 0.68$	$D_{\mathrm{IPL}}=0.88\pm 0.04$
(b) : R=0.8 cm	$L_{\theta =164}=0.22$	$D_{\mathrm{IPL}}=0.17$	$0.86\leq L_{\theta =111}\leq 0.95$	$D_{\mathrm{IPL}}=0.92\pm 0.04$
(c) : R=1.0 cm	$L_{\theta =168}=0.20$	$D_{\mathrm{IPL}}=0.17$	$0.82\leq L_{\theta =127}\leq 0.95$	$D_{\mathrm{IPL}}=0.88\pm 0.04$
(d) : R=2.0 cm	$L_{\theta =174}=0.20$	$D_{\mathrm{IPL}}=0.17$	$0.69\leq L_{\theta =156}\leq 0.97$	$D_{\mathrm{IPL}}=0.88\pm 0.04$

It is clear that most of the vertical and azimuthal results in each section have the same order of magnitude, with the mismatch falling within the standard deviation of the results. Moreover, a clear consistency of the vertical IPL point for all the different radius exists, which matches the IPL theory found for half-infinity surfaces, as said before. It should be noted that the range obtained for the MC $L_{\mathrm{IPL}}$ arises from its sinusoidal dependence on the azimuthal STD results that exhibit a constant STD of ±4  deg.

Experimentally, the IPL points were measured at an azimuthal angle of 144 deg±3 deg⁡ and a longitudinal distance of 0.33±0.05  cm. The differences between theoretical and experimental results have previously been attributed to averaging effects caused by the detector size and the distance from the air–tissue interface.^19^^,^^21^^,^^22^^,^^24^ Using Eq. (20), the arc length was calculated as ∼0.31  cm, within the range of [0.28 − 0.33], which agrees well with the vertical measurement of 0.33 cm and falls within the error margin. Thus, although theory, MC simulations, and experiments yield slightly different coordinates, they consistently identify the same behavior.

Naturally, as with any experimental setup, limitations such as pixel resolution, imperfections in the phantom geometry, laser spot alignment, and the endoscope angle introduce systematic offsets. Nevertheless, the presence of the IPL point was consistently confirmed.

Furthermore, the IPL allowed us to calculate the absorption coefficient of an *ex vivo* tissue (chicken breast), yielding a value of $\mu_{\text{a}}=0.94\;{\mathrm{cm}}^{-1}$. This result falls within the range reported in the literature.^19^^,^^21^

Except for the discrepancy between the theoretical results and the experimental, the differences in general between theory, MC simulations, and experiments stem from several factors. First, phantom parameters such as anisotropy and absorption are not precisely known, so simulations cannot perfectly reproduce the experimental conditions. Second, the diffusion theory assumes homogeneous, infinite tissue with ideal boundary conditions, which differs from both MC simulations and experimental phantoms. Finally, both MC and experimental setups use finite-size detectors, a factor not considered in the theoretical model.

From a practical perspective, the longitudinal IPL point is likely the most useful in endoscopy. Modern medical endoscopes include ports for external light sources; thus, by orienting the endoscope at 90 deg toward the esophageal wall, it is possible to measure the longitudinal IPL point directly. Measuring the azimuthal IPL point is also possible in this configuration but requires more advanced image processing to correct for curvature.

The next step will be to evaluate the IPL point for diagnostic purposes. In particular, blood absorption differences between benign and malignant tissues may serve as an indicator for pathology. Using the IPL point as a reference, as previously proposed,^12^ absorption can be assessed independently of scattering, enabling deeper examination of tissues such as the esophageal wall. In the future, this approach may provide a powerful tool for detecting polyps and other pathologies at greater tissue depths.

## Data Availability

All data in support of the findings of this paper are available within the article.
